# Global research hotspots and trends in brucellosis: a bibliometric and visualization analysis based on CiteSpace and VOSviewer

**DOI:** 10.3389/fmed.2025.1617217

**Published:** 2025-11-17

**Authors:** Wen Liu, Yonghai Dong, Weidong Zhou, Aimeng Sun

**Affiliations:** 1 Young Scientific Research and Innovation Team, Ji‘an Municipal Center for Disease Control and Prevention, Ji'an, Jiangxi, China; 2 Young Scientific Research and Innovation Team, Jiangxi Provincial Center for Disease Control and Prevention, Nanchang, Jiangxi, China

**Keywords:** brucellosis, bibliometrics, visualization analysis, CiteSpace, VOSviewer

## Abstract

**Objective:**

This study aims to elucidate the global research dynamics of brucellosis through bibliometric visualization analysis. The findings have major implications for advancing academic progress in the field of brucellosis research, fostering interdisciplinary collaboration, and strengthening disease prevention and control.

**Methods:**

The Web of Science Core Collection database was utilized as the data source for this study, and literature published between January 1, 1901, and December 31, 2024 was retrieved. CiteSpace and VOSviewer software tools were employed to conduct a visual analysis of the publication volume, countries, institutions, authors, journals, and keywords.

**Results:**

A total of 12,810 articles were included in this analysis, with the first publication dating back to 1901.The annual publication volume has increased over the years. The United States had the highest volume of publications and intermediary centrality. The United States Department of Agriculture (USDA) and the journal *Infection and Immunity* were found to be the most influential. Professors Kim S. and Pappas G. have made the most significant contributions to the field of brucellosis. Keyword analysis indicated that the top five high-frequency keywords were “*brucella abortus*,” “infection,” “*brucella melitensis*,” “diagnosis,” and “cattle.” Among the five formed clusters, cluster #0 (virulence) was larger and newer, with persisting research hotspots. In the keyword burst analysis, the keyword “elisa” exhibited the highest burst strength of 41.47. Recent emerging keywords include “one health,” “risk factors,” and “seroprevalence.”

**Conclusion:**

This study indicates that brucellosis research is predominantly concentrated in developed countries such as the United States. Professor Pappas is a key contributor in this research area. The “one health” approach to brucellosis is a current research hotspot. Based on these findings, future studies focused on content, methods, and value may represent a new trend in brucellosis research.

## 1 Introduction

Brucellosis is a zoonotic infectious disease caused by various species of the genus *Brucella* (1), which can affect humans and several other animal species. The *Brucella* species of significant zoonotic importance include *Brucella abortus, Brucella melitensis, Brucella suis*, and *Brucella canis* (2). The World Health Organization (WHO) has classified brucellosis as one of the world's major neglected zoonotic diseases. The disease primarily spreads through contact with infected animals or animal products, as well as the consumption of fresh, unpasteurized dairy products (3). Although rare, human-to-human transmission can occur (4), posing occupational hazards to individuals such as livestock farmers, dairy workers, slaughterhouse workers, and veterinarians (5, 6). Typically, brucellosis manifests as non-specific clinical symptoms, with patients in the acute phase mainly experiencing fever, fatigue, muscle and joint pain, and enlargement of the liver, spleen, and lymph nodes (7). If the infection persists, it may lead to neurological complications, including paravertebral abscesses and spinal epidural abscesses (8). Furthermore, it can cause sequelae such as uveitis (9).

In recent years, brucellosis has rapidly spread to over 170 countries and regions. Current research indicates that there may be up to 2.1 million new cases annually worldwide (10), imposing a substantial burden on the health systems and severely affecting economic development at the individual, community, and national levels. Current research hotspots and directions in brucellosis focus on pathogenic mechanisms, novel diagnostic and preventive technologies, and associated challenges. Understanding its pathogenic mechanisms requires deciphering its key immune evasion strategies, including how the Type IV Secretion System (T4SS) secretes the effector protein VirB, modifies lipopolysaccharide (LPS) to suppress host immunity, and enables long-term survival within macrophages. Moreover, the role of immune checkpoint molecules such as PD-1/CTLA-4 in T-cell exhaustion during chronic infections provides novel targets for immunotherapy (11, 12). Innovations in diagnostic technologies aim to overcome the limitations of traditional serological methods. Emerging molecular diagnostic techniques (e.g., ELISA kits targeting the VirB12 protein, nanopore sequencing, and qPCR/LAMP) and AI-based deep neural network models are driving improvements in detection sensitivity, specificity, and early risk prediction (11, 13, 14). In terms of treatment and vaccine development, to address the high recurrence rate following standard antibiotic therapies, nanoparticle-based antibiotic delivery systems and phage cocktail therapies are being explored. In the vaccine field, next-generation vaccines such as RS bivalent vaccines and ΔvirB12 gene-deleted strains constructed via CRISPR-Cas9 are under development (11, 15). The main challenges include the lack of diagnostic standardization (only 16% of studies on brucellosis diagnosis in livestock adhere to the WOAH guidelines), as well as obstacles to prevention and control posed by the absence of human vaccines and antibiotic resistance (14, 16).

Currently, academic research on brucellosis is becoming increasingly diversified, particularly given the substantial changes in the global epidemiology of brucellosis over the past few decades. Thus, summarizing and analyzing the current status, hotspots, and trends in brucellosis research is essential. Bibliometrics is a methodology that effectively displays the current state and trends of a specific research topic or field through visualization (17), utilizing visual software tools like CiteSpace and VOSviewer. This study employs CiteSpace and VOSviewer to perform a visual analysis of the literature related to brucellosis sourced from the Web of Science Core Collection database. The aim is to outline the historical development of this research field, identify prevalent keywords and core themes, clarify research distribution and collaborative relationships, highlight research hotspots and trends, and reveal emerging frontiers in the field. The findings of this study are crucial for promoting academic progress in the field, facilitating interdisciplinary collaboration, and enhancing efforts in disease prevention and control.

## 2 Materials and methods

### 2.1 Inclusion and exclusion criteria

The literature included in this study was retrieved from the Science Citation Index Expanded and Social Sciences Citation Index within the Web of Science (WoS) Core Collection. The literature meeting the following criteria was included: (1) Research related to brucellosis; (2) Publication dates ranging from January 1, 1901 to December 31, 2024. The following types of literature were excluded: (1) Meeting Abstract, (2) Proceeding Paper, (3) Letter, (4) Editorial Material, (5) Note, (6) Correction, (7) Early Access, (8) Book Chapters, (9) Correction, Addition, (10) Abstract of Published Item, (11) Book Review, (12) Data Paper, (13) News Item, (14) Retracted Publication, (15) Discussion, (16) Reprint, (17) Biographical-Item, (18) Retraction, (19) Item About an Individual.

### 2.2 Literature retrieval

**Figure 1:**
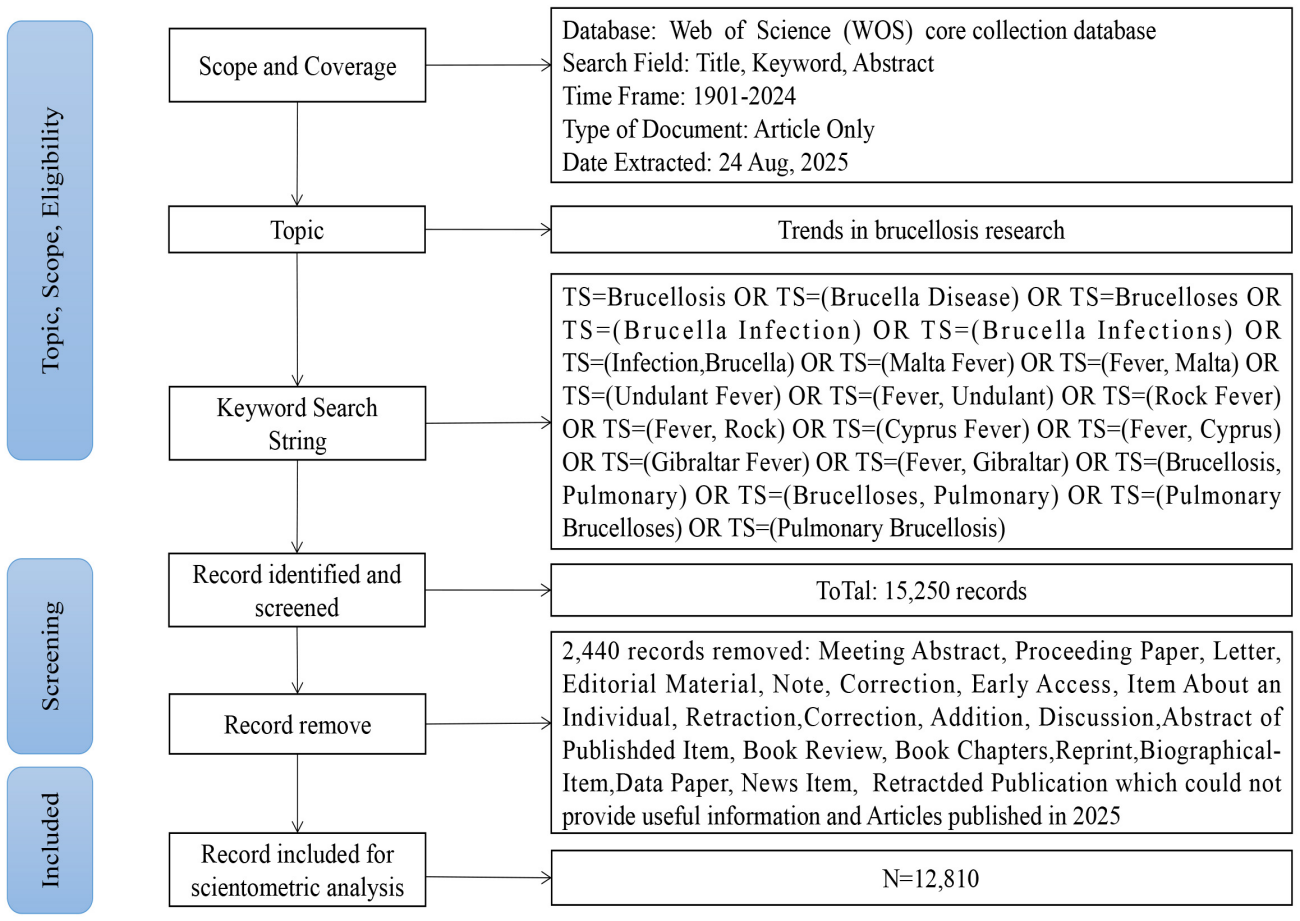
The research flow chart.

The search formula was: *TS* = *Brucellosis OR TS* = *(Brucella Disease) OR TS* = *Brucelloses OR TS* = *(Brucella Infection) OR TS* = *(Brucella Infections) OR TS* = *(Infection, Brucella) OR TS* = *(Malta Fever) OR TS* = *(Fever, Malta) OR TS* = *(Undulant Fever) OR TS* = *(Fever, Undulant) OR TS* = *(Rock Fever) OR TS* = *(Fever, Rock) OR TS* = *(Cyprus Fever) OR TS* = *(Fever, Cyprus) OR TS* = *(Gibraltar Fever) OR TS* = *(Fever, Gibraltar) OR TS* = *(Brucellosis, Pulmonary) OR TS* = *(Brucelloses, Pulmonary) OR TS* = *(Pulmonary Brucelloses) OR TS* = *(Pulmonary Brucellosis)*. A total of 15,250 articles were retrieved between January 1, 1901, and December 31, 2024, specifically filtering for article types classified as “article” and “review article,” which resulted in the exclusion of 2,404 articles. The data were downloaded in full record plain text format, and duplicates were assessed using CiteSpace, revealing no duplicates. After excluding 36 articles published in 2025, the final analysis included 12,810 articles. The literature retrieval process is illustrated in Figure 1. This study focuses on a bibliometric analysis of published literature and does not involve any human or animal experiments, rendering ethical review unnecessary.

### 2.3 Statistical analysis

Annual and cumulative publication trends were plotted using Microsoft Office Excel 2016. The data were imported into CiteSpace (V6.2.R6) for analysis of countries, institutions, keyword clustering, and burst keywords. In the CiteSpace visualization, nodes represent elements such as countries, institutions, and keywords, lines indicate the strength of collaboration between these nodes and the purple outer circle represents intermediary centrality, which is used to assess the importance and influence of specific nodes; cluster labels represent the relevance of keywords within clusters, with two key metrics being modularity (*Q* value) and silhouette coefficient (*S* value). Generally, a *Q* value greater than 0.3 indicates significant clustering, while an *S* value exceeding 0.5 is often regarded as reasonable. VOSviewer (V1.6.20) was utilized to analyze journals, authors, and co-citation of articles, where nodes represent elements such as journals, authors, and articles. The size of each node corresponds to publication volume or citation degree, and the thickness of lines represents the degree of collaboration between nodes.

## 3 Results

### 3.1 Analysis of annual publications

**Figure 2:**
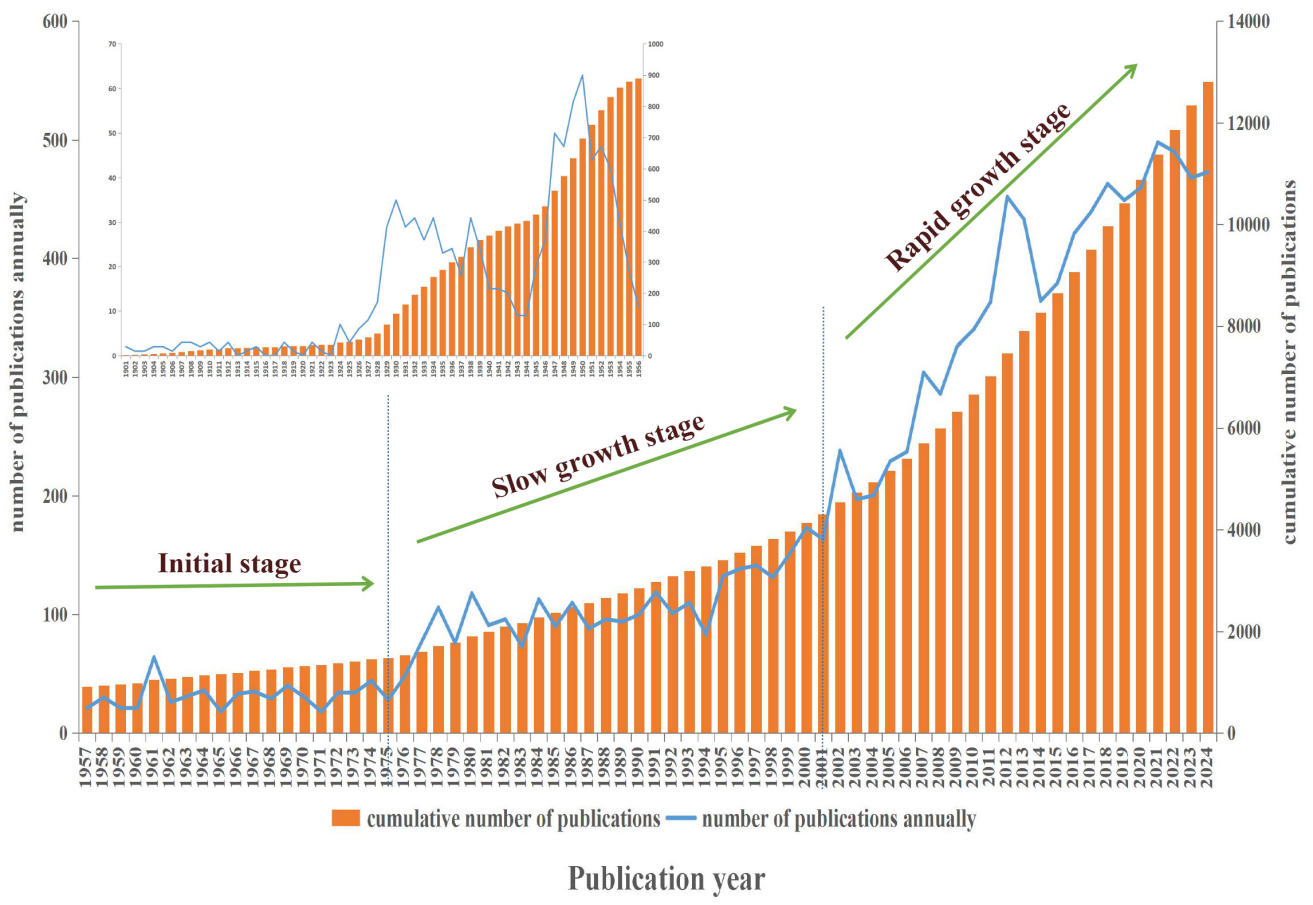
Annual and cumulative trend of publications.

The analysis of annual publication output reveals the temporal variation in the number of literatures, facilitating the identification of research hotspots and development trends. This study included a total of 12,810 valid articles, with the earliest publication dating back to 1901. The volume of publications remained in single digits for the subsequent 27 years. Between 1928 and 1956, publication numbers exhibited significant fluctuations; however, they largely remained low, reflecting limited attention to this field during its formative years. From 1957 to 1975, the annual publication volume stabilized at a low level. Between 1976 and 2001, a consistent upward trend in publication volume emerged, indicating increasing interest in this field. After 2002, the annual publication volume increased rapidly, consistently surpassing 400 articles each year over the past 5 years, which indicates significant research progress in this field, as detailed in Figure 2.

### 3.2 Analysis of national, institutional, and author collaborations

#### 3.2.1 Analysis of national publication volume and collaboration

**Figure 3:**
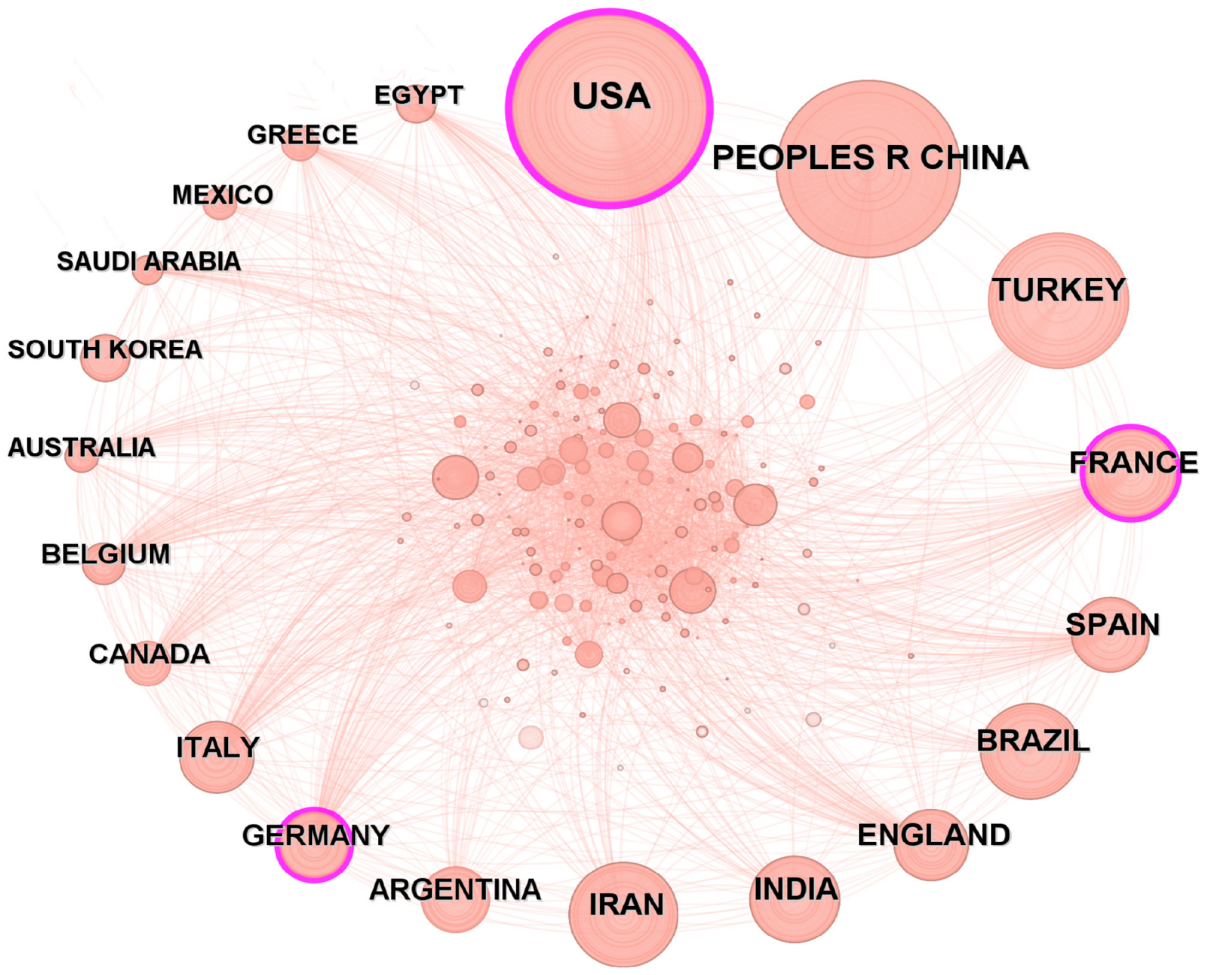
Countries co-occurrence analysis.

The analysis of national publication output and collaborative relationships enables a macroscopic understanding of the global development landscape in the research field, uncovers the global research pattern, identifies core cooperative networks, and provides valuable guidance for scientific research collaboration and resource allocation. Researchers from 168 countries conducted studies on brucellosis, with the United States leading at 18.51% (2,371 articles), followed by China at 8.42% (1,079 articles) and Turkey at 6.55% (839 articles). The countries with higher intermediary centrality scores include the United States (0.28), France (0.15), and Germany (0.10), while the remaining countries had centrality scores less than 0.10, as shown in Table 1. Figure 3 illustrates the collaboration among countries, with the analysis indicating that the United States maintains a prominent position in the research field of brucellosis and has close collaborations with other countries.

**Table 1:** Top 10 Countries in terms of number of publications and intermediary centrality.

**Rank**	**Publication**			**Centrality**	
	**Number**	**Percent (%)**	**Country**	**Centrality**	**Country**
1	2,371	18.51	USA	0.28	USA
2	1,079	8.42	Peoples R China	0.15	France
3	839	6.55	Turkey	0.10	Germany
4	646	5.04	France	0.09	England
5	581	4.54	Spain	0.08	Belgium
6	530	4.14	Brazil	0.07	Switzerland
7	501	3.91	England	0.07	South Africa
8	495	3.86	India	0.05	Italy
9	480	3.75	Iran	0.05	Canada
10	407	3.18	Argentina	0.05	Netherlands

#### 3.2.2 Analysis of institutional publication volume and collaboration

**Figure 4:**
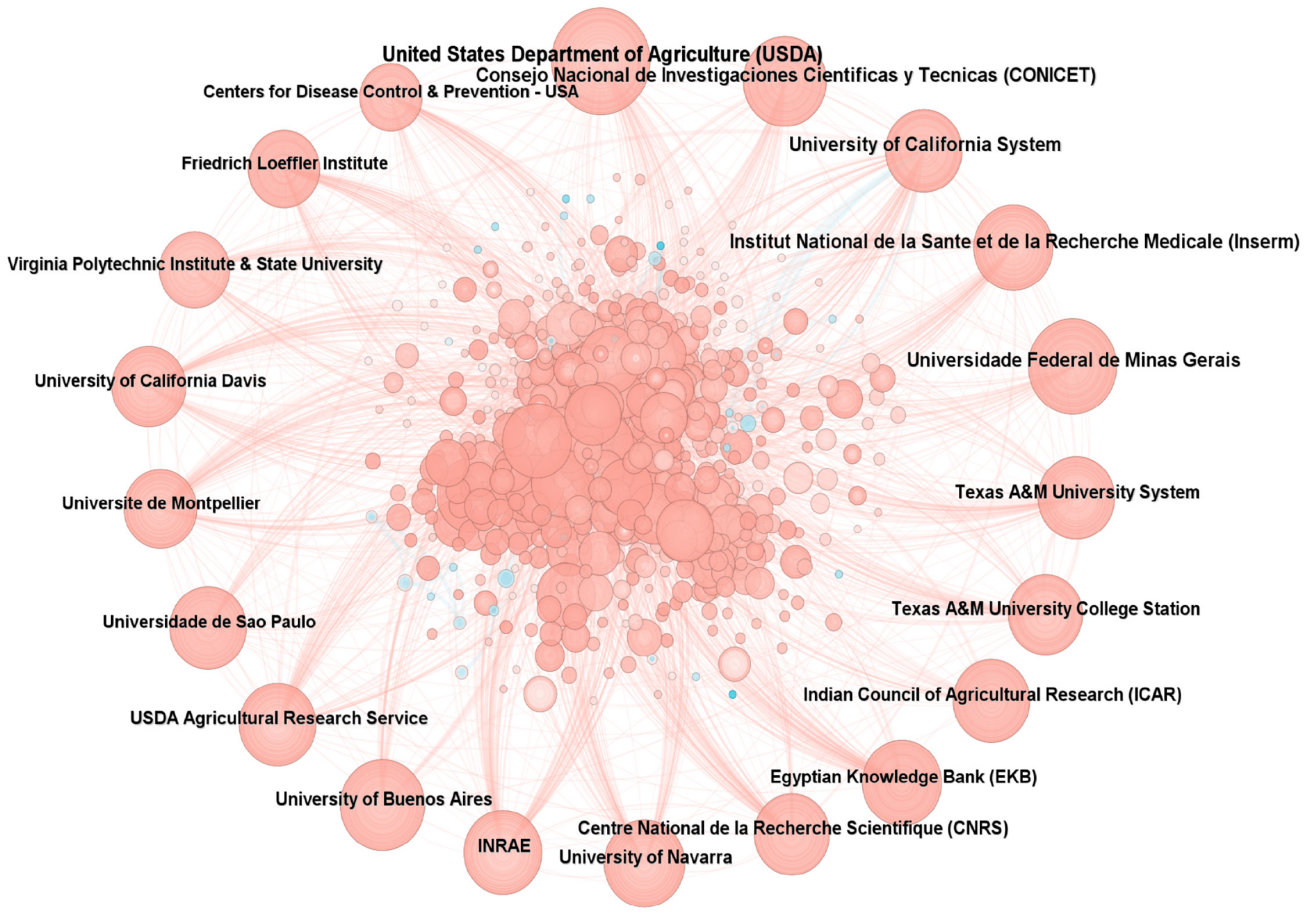
Cooperative network diagram of research institutions.

The analysis of research institutions serves as a crucial indicator for evaluating research achievements. It facilitates the rapid identification of the most active and impactful research institutions in the field, clearly illustrates the cooperation models and intensity among institutions, and sheds light on the developmental dynamics of the entire research domain. A total of 9,100 institutional researchers had engaged in studies on brucellosis, with the top three institutions by publication volume being the United States Department of Agriculture (USDA), Consejo Nacional de Investigaciones Cientificas y Tecnicas (CONICET), and the University of California System. None of the intermediary centrality scores exceeded 0.10, as shown in Figure 4, which illustrates the collaborative relationships between institutions. The results indicate that the USDA holds a significant position in the field of brucellosis research and maintains close collaborations with other institutions.

#### 3.2.3 Analysis of author publication volume and co-citation

**Figure 5:**
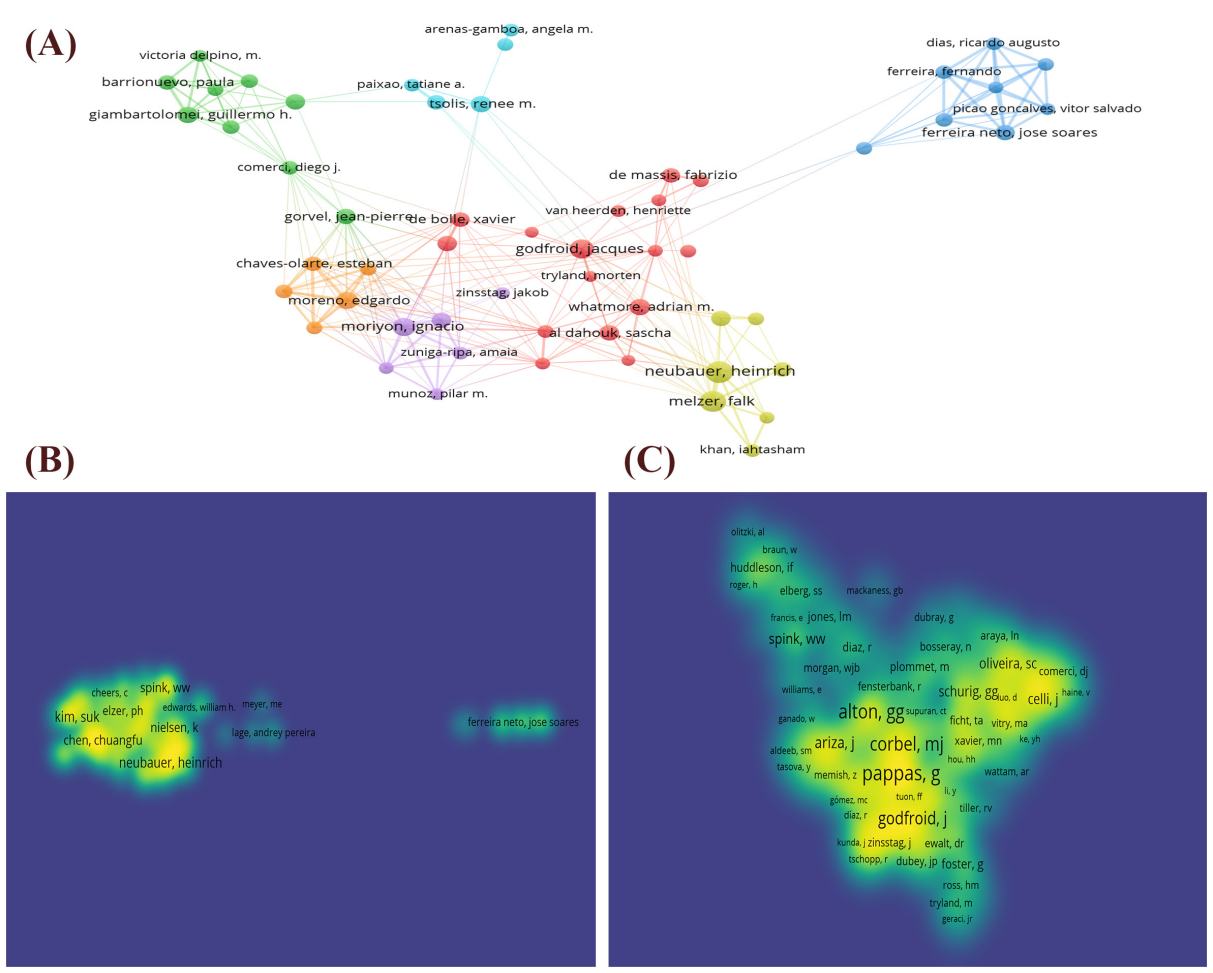
Author cooperation network map. **(A)** Network visualization of co-authorship of the authors. **(B)** Density visualization of citation of authors. **(C)** Density visualization of co-citation of authors.

The analysis of authors' publication output and co-citation helps to quickly identify key authors and pivotal research findings, contributing to the elucidation of research topics and results, as well as tracking research frontiers. In total, 42,351 authors conducted research on brucellosis. Based on the criterion of having published at least 20 articles, we selected 122 authors for visualization. A collaborative network diagram (Figure 5A) was created to illustrate the publication volume and collaboration relationships among these authors. Figures 5B, C illustrate the citation densities of 521 cited authors with at least 10 publications and 861 co-cited authors with a minimum of 50 citations, respectively. Table 2 displays the top 10 authors ranked by publication volume and co-citation frequency. Notably, Professor Kim S. has the highest publication volume with 78 articles, while Professor Pappas G. leads in co-citation frequency (2,456 times). This indicates that these two researchers have made significant contributions to the field of brucellosis research, which has received widespread recognition.

**Table 2:** Top 10 authors and co-cited authors.

**Rank**	**Author**		**Co-cited author**	
	**Number**	**Author**	**Number**	**Co-cited author**
1	78	Kim S.	2,456	Pappas G.
2	75	Neubauer H.	2,178	Alton G.G.
3	66	Melzer F.	1,907	Corbel M.J.
4	57	Godfroid J.	1,445	Godfroid J.
5	56	Spink W.W.	1,378	Young E.J.
6	56	Nielsen K.	1,154	Cloeckaert A.
7	54	Chen C.F.	1,138	Nielsen K.
8	53	Moriyon I.	990	Ariza J.
9	52	Min W.	941	Moreno E.
10	51	Cloeckaert A.	879	Spink W.W.

### 3.3 Analysis of journal distribution and co-citation

**Figure 6:**
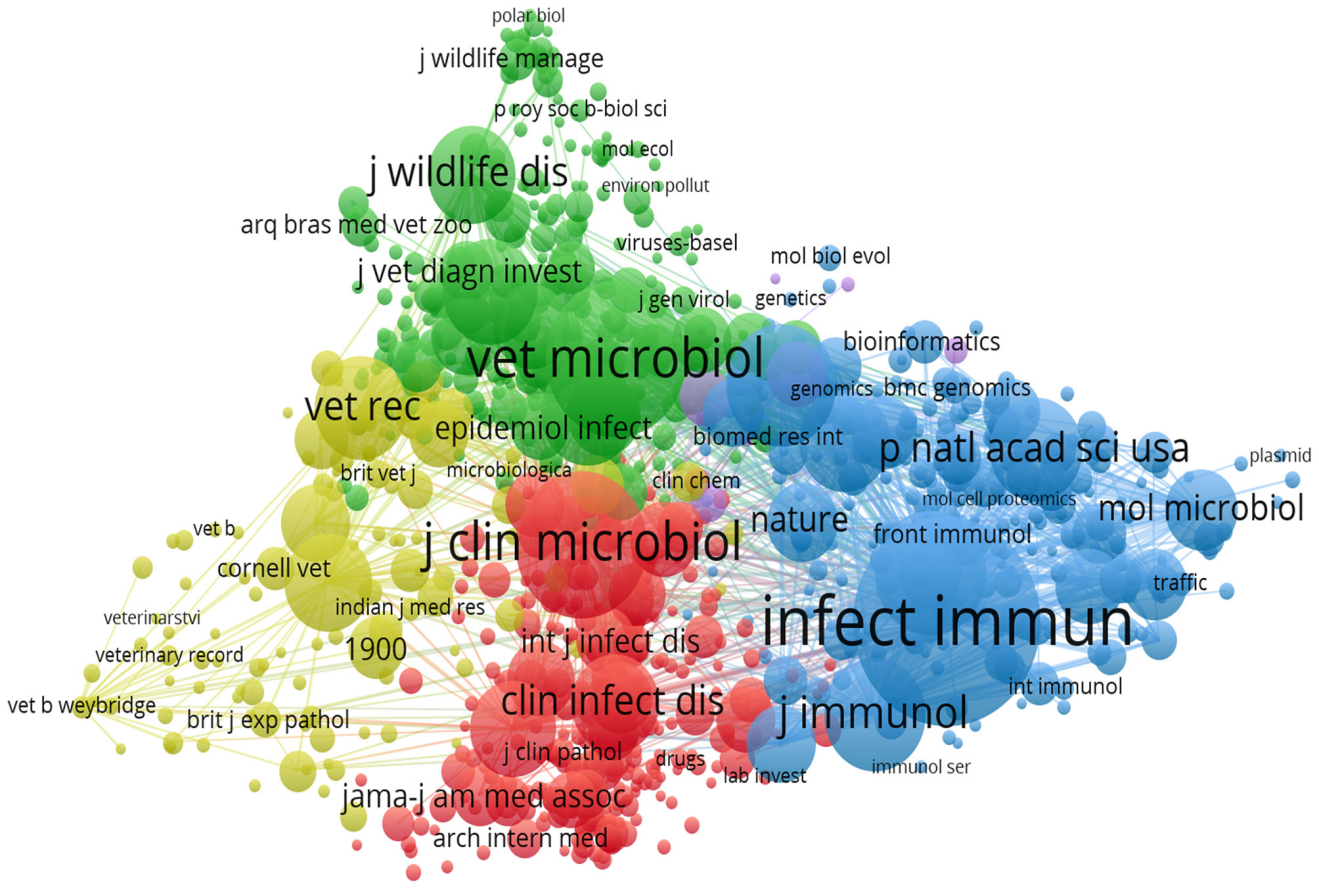
Co-citation map of journals.

The analysis of journal distribution and co-citation aims to identify the most influential platforms in brucellosis research, thereby assisting researchers in selecting the most active and impactful journals to maximize the visibility and impact of their research outcomes. A total of 1,936 journals had published literature pertaining to brucellosis, with the top 10 journals mainly covering disciplines such as infection and immunity, veterinary science, wildlife, vaccines, and clinical microbiology (Table 3). In 2024, the Journal Citation Reports (JCR) are predominantly concentrated in zones 1 and 2, all exhibiting impact factors (IF) below 10. The top 10 most cited journals have each received more than 4,000 citations, the journal with the highest citations being *Infection and Immunity* (16,026 times), while the journal with the highest IF is the *Proceedings of the National Academy of Sciences of the United States of America* (IF = 9.1). Utilizing a minimum citation threshold of 50, a visual co-citation network of journals was generated using VOSviewer (Figure 6), revealing a positive co-citation relationship among journals such as *Infection and Immunity, Journal of Clinical Microbiology*, and *Veterinary Microbiology*.

**Table 3:** Top 10 journals and co-cited journals.

**Rank**	**Journal**			**Cited Journal**		
	**Number**	**IF (Q)**	**Journal**	**Citations (** ***n*** **)**	**IF (Q)**	**Cited journal**
1	282	2.8 (Q2)	Infection and Immunity	16,026	2.8 (Q2)	Infection and Immunity
2	246	2.7 (Q1)	Veterinary Microbiology	9,101	2.7 (Q1)	Veterinary Microbiology
3	210	2.6 (Q2)	PLoS ONE	8,035	5.4 (Q1)	Journal of Clinical Microbiology
4	204	1.2 (Q3)	Journal of Wildlife Diseases	5,711	3.0 (Q3)	Journal of Bacteriology
5	192	2.4 (Q1)	Preventive Veterinary Medicine	5,475	2.6 (Q2)	PLoS ONE
6	161	1.7 (Q2)	Tropical Animal Health and Production	4,688	3.4 (Q2)	Journal of Immunology
7	153	1.4 (Q2)	American Journal of Veterinary Research	4,172	2.4 (Q1)	Preventive Veterinary Medicine
8	145	5.4 (Q1)	Journal of Clinical Microbiology	4,063	6.6 (Q1)	Emerging Infectious Diseases
9	137	1.8 (Q2)	Journal of the American Veterinary Medical Association	4,042	9.1 (Q1)	Proceedings of the National Academy of Sciences of the United States of America
10	136	3.5 (Q2)	Vaccine	4,011	1.4 (Q2)	Veterinary Record

### 3.4 Analysis of co-cited literature

By quantifying the citation relationships between literatures, literature co-citation analysis enables researchers to efficiently map the knowledge structure of the research field, identify core literatures, and discover research hotspots and cutting-edge directions. This provides a clear roadmap for subsequent studies and plays a vital role in guiding future research directions. In the literature on brucellosis research, there are a total of 200,197 references, with the top 10 most cited works listed according to citation frequency. Each of these 10 co-cited references has received over 300 citations, primarily addressing topics such as comprehensive reviews of brucellosis, human brucellosis, laboratory techniques for brucellosis, and the epidemiological distribution of the disease. The most frequently cited work is “*The New Global Map of Human Brucellosis*” authored by Pappas G. et al. in (21), which has accrued a total of 1,129 citations, as listed in Table 4.

**Table 4:** Top 10 most co-cited research articles.

**Rank**	**Title**	**Source**	**Publication year**	**First author**	**Citations (*n*)**
1	The new global map of human brucellosis	Lancet Infectious Diseases	2006	Pappas G.	1,129
2	Brucellosis: an Overview	Emerging Infectious Diseases	1997	Corbel M.J.	750
3	Brucellosis	New England Journal of Medicine	2005	Pappas G.	673
4	Techniques for the Brucellosis Laboratory	Institute National de la Recherche Agronomique	1988	Alton G.G.	623
5	Human brucellosis	Lancet Infectious Diseases	2007	Franco M.P.	589
6	An overview of human brucellosis	Clinical Infectious Diseases	1995	Young, E.J.	436
7	Brucellosis:a re-emerging zoonosis	Veterinary Microbiology	2010	Seleem M.N.	386
8	From the discovery of the Malta fever's agent to the discovery of a marine mammal reservoir, brucellosis has continuously been a re-emerging zoonosis	Veterinary Research	2005	Godfroid J.	318
9	Differentiation of Brucella abortus bv. 1, 2, and 4, Brucella melitensis, Brucella ovis, and Brucella suis bv. 1 by PCR	Journal of Clinical Microbiology	1994	Bricker B.J.	317
10	Complications Associated with Brucella melitensis Infection: A Study of 530 Cases	Medicine	1996	Colmenero J.D.	315

### 3.5 Keyword analysis

#### 3.5.1 Co-occurrence analysis

**Figure 7:**
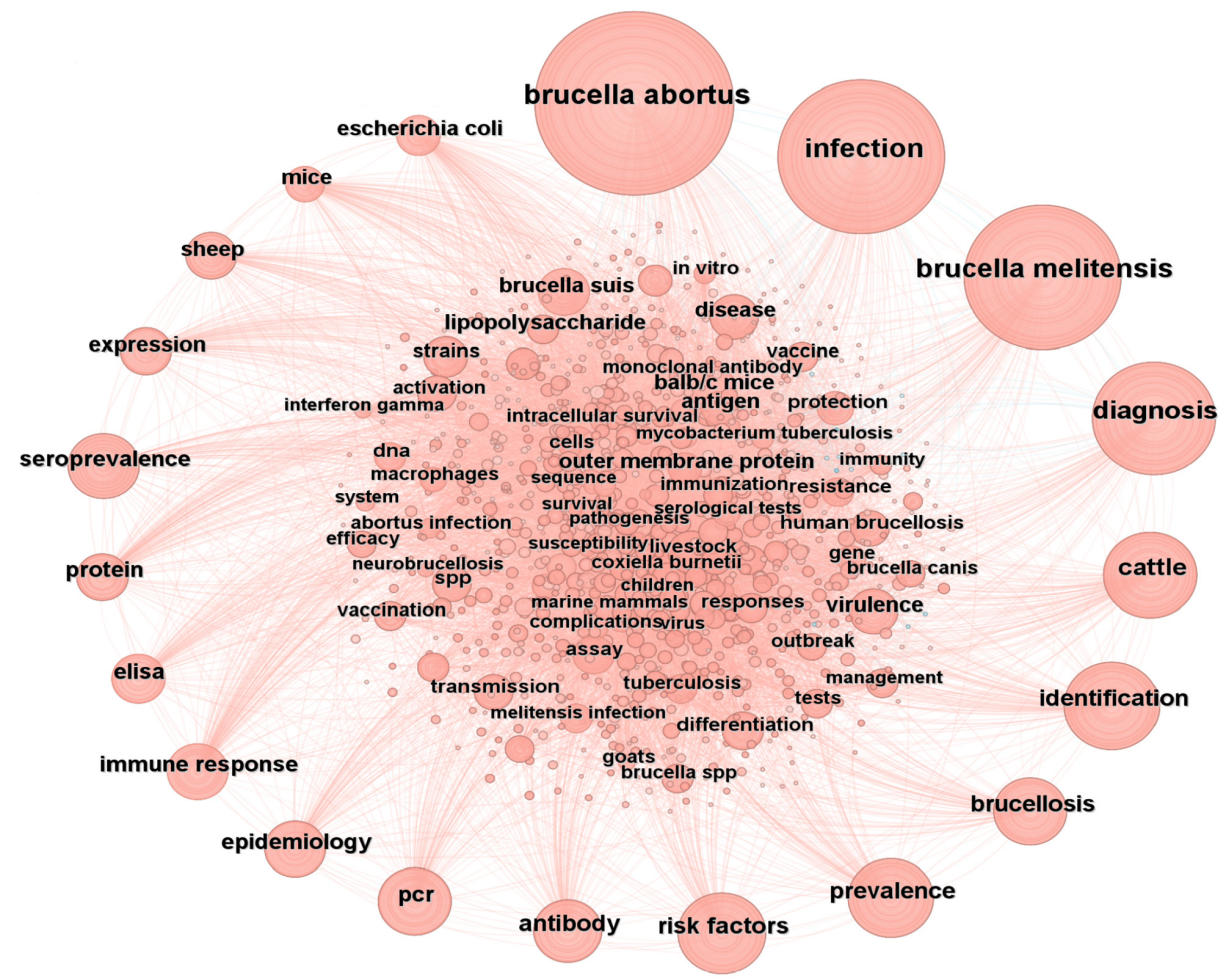
Keywords contribution analysis.

Keyword co-occurrence analysis visually demonstrates the relationships between various keywords and highlights the connections within the entire research field. CiteSpace was used to conduct a co-occurrence analysis of the keywords in the 12,810 included documents. By merging equivalent terms, such as “brucellosis” and “q fever,” “pcr” and “polymerase chain reaction,” as well as “*brucella abortus*” and “*abortus*,” a total of 1,060 relevant keywords were identified (Figure 7). The results indicated that the intermediary centrality of all keywords did not exceed 0.10. The top 20 keywords ranked by frequency are presented in Table 5, with “*brucella abortus*,” “infection,” “*brucella melitensis*,” “diagnosis,” and “cattle” comprising the top five positions. This indicates that “*brucella abortus*” is the most frequently used keyword and holds significant value in the research area of brucellosis.

**Table 5:** Top 20 high-frequency keywords.

**Rank**	**Count**	**Keyword**	**Rank**	**Count**	**Keyword**
1	2,353	*Brucella abortus*	11	475	PCR
2	1,739	Infection	12	427	Epidemiology
3	1,575	*Brucella melitensis*	13	418	Immune response
4	1,064	Diagnosis	14	373	ELISA
5	698	Cattle	15	352	Protein
6	665	Identification	16	344	Seroprevalence
7	579	Brucellosis	17	338	Expression
8	557	Prevalence	18	316	Sheep
9	522	Risk factors	19	314	Mice
10	513	Antibody	20	302	*Escherichia coli*

#### 3.5.2 Cluster analysis

**Figure 8:**
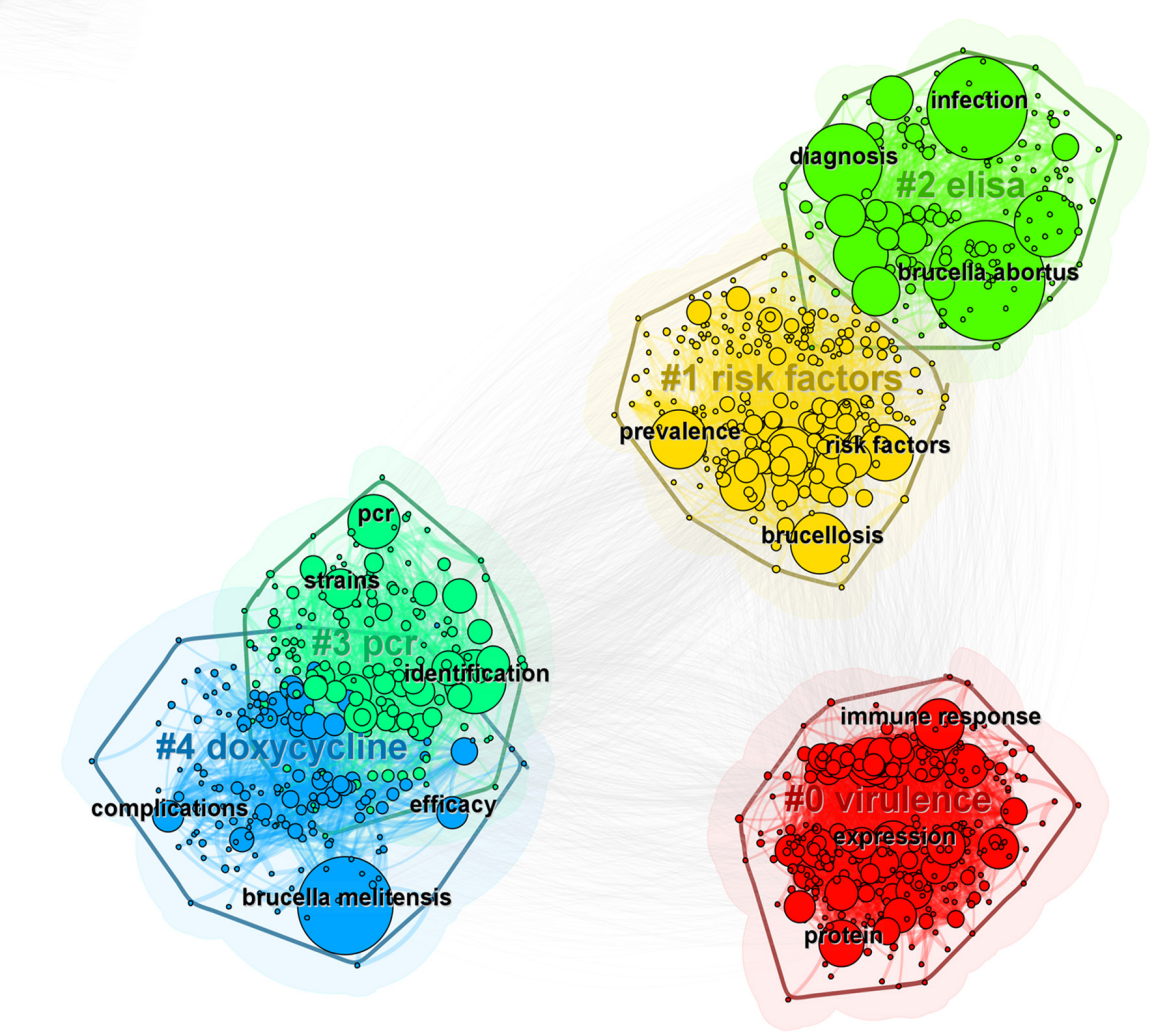
Cluster map of keywords.

Keyword clustering effectively illustrates the relationships among keywords within a research domain, identifies correlations between different research themes, and reveals their internal structures and hotspots. In this study, a keyword clustering diagram was constructed in CiteSpace (Figure 8), which primarily presents five major clusters: #0 (virulence), #1 (risk factors), #2 (elisa), #3 (pcr), and #4 (doxycycline). Furthermore, the top three frequently used keywords within each cluster are highlighted, with overlaps observed in the clusters of “risk factors” and “elisa,” as well as “pcr” and “doxycycline.” This overlap indicates the presence of cross-integration among these research areas.

#### 3.5.3 Timeline and burst word analysis

**Figure 9:**
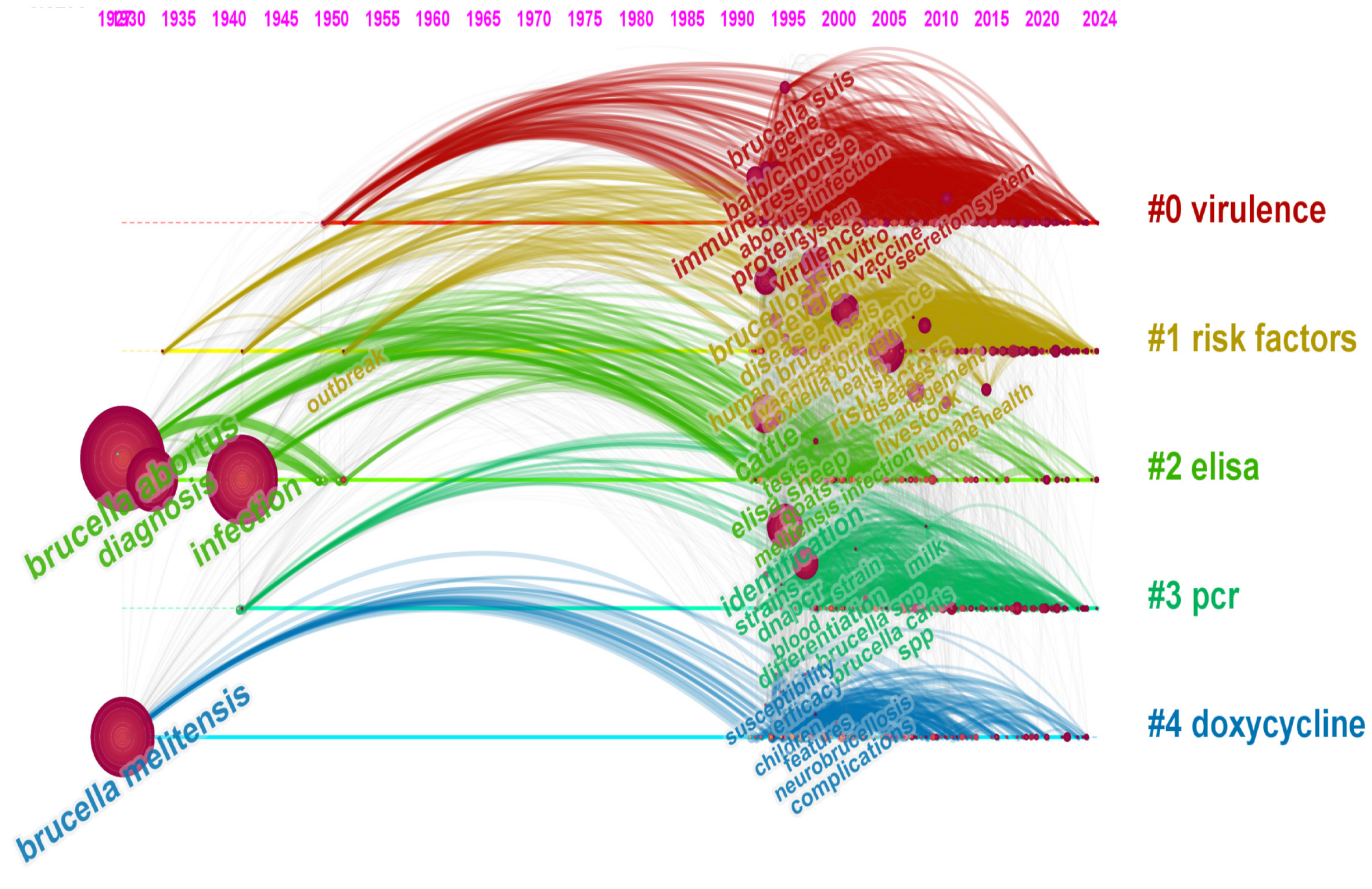
Keyword timeline distribution.

**Figure 10:**
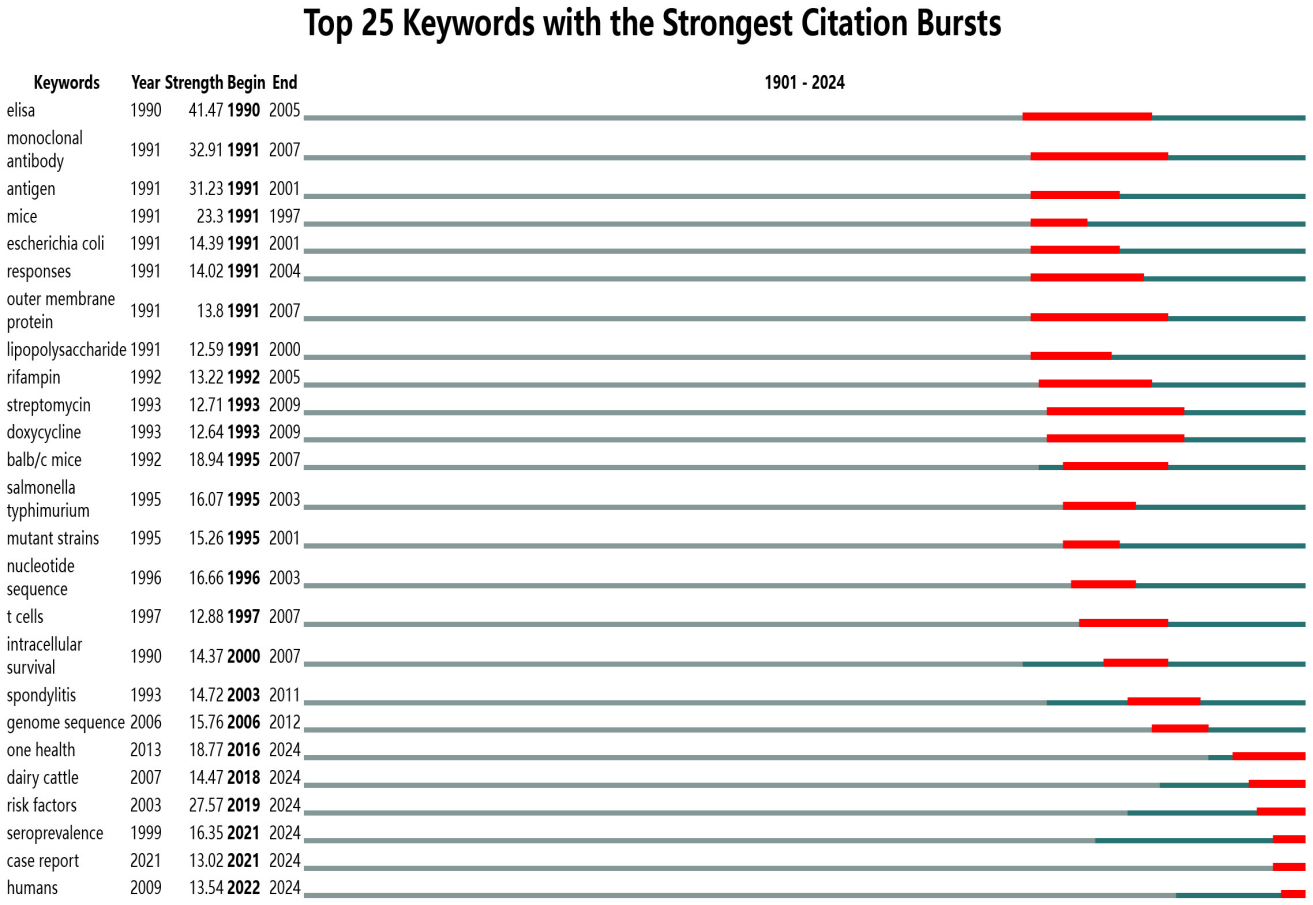
Map of top 25 keywords with the strongest citation bursts.

The utilization of keyword clustering in this study to construct a timeline of keywords (Figure 9) facilitates an intuitive depiction of the evolution and interrelationships of hotspots such as “virulence,” “risk factors,” and “elisa,” thereby enhancing predictions regarding future research directions in brucellosis. The burst detection function of CiteSpace elucidates research hotspots and trends within the academic domain, forecasting potential frontiers and emerging focal points. Figure 10 illustrates the top 25 keywords with the strongest citation bursts in brucellosis research, providing details on the keywords, their inaugural appearance, burst intensity, and the duration in which these keywords became hotspots. The higher the burst strength value, the higher the popularity of that term in that timeframe, with “elisa” having the highest burst strength of 41.47. Between 1990 and 2012, the principal keywords included “elisa,” “monoclonal antibody,” and “outer membrane protein,” all of which maintained prominence for over 15 years. From 2016 to 2024, the focus of emerging keywords progressively shifted toward “one health,” “risk factors,” and “seroprevalence”.

## 4 Discussion

This study employs bibliometric methods to analyze global research literature on brucellosis over the past century, aiming to comprehensively showcase the development trends, current state of research, and hotspots in this field. Through literature retrieval, three similar studies were identified (18–20), which focused primarily on the clinical symptoms of brucellosis. In contrast, our study aims to provide a comprehensive and in-depth analysis of the field of brucellosis.

According to publication volume data, the first document recorded in the WoS Core Collection database was published in 1901. Since 2002, the volume of publications has significantly increased, which is correlated with the rapid spread of brucellosis worldwide. In 1973, outbreaks were reported in 75 countries globally, and by 2008, this number had risen to over 160 countries. Currently, more than 170 countries and regions are affected. Recent research indicates that the annual incidence of new cases worldwide may reach 2.1 million (10).

A total of 168 countries have researchers engaged in the study of brucellosis, with the combined publication volume from the United States, China, and France accounting for nearly one-third of the total. Intermediary centrality is primarily concentrated in the United States (0.28), reflecting its significant international influence. Although China ranks second in publication volume, its intermediary centrality measures less than 0.10, indicating limited international collaboration and constraints on the effectiveness of research dissemination. Among the 9,100 institutions, the USDA ranks first, with a publication volume of 271 articles, demonstrating its extensive collaborative network and notable influence. The journals publishing these works are predominantly situated in the first and second quartiles, with all IFs below 10. The cited literature constitutes the knowledge base of this research field. Notably, the paper by Pappas et al. (21) was cited 2,456 times, while a single article reached 1,129 citations, underscoring its authority in the field and fostering further development of the research area.

Keywords effectively reflect the themes of scholarly literature from various perspectives. Analyzing keywords within the literature enhances the understanding of the research field, its hotspots, and directions. The co-occurrence analysis reveals that the top 5 most frequently cited keywords are “*brucella abortus*,” “infection,” “*brucella melitensis*,” “diagnosis,” and “cattle.” The keyword clustering diagram revealed a modularity *Q* value of 0.40 and a silhouette coefficient *S* value of 0.77, demonstrating good independence and consistency in clustering (22–24). The top 5 clusters, “virulence,” “risk factors,” “elisa,” “pcr,” and “doxycycline,” underscore the significance of these fields in brucellosis research. The overlap of clusters among “risk factors” and “elisa,” as well as “pcr” and “doxycycline,” suggest intersections and integrations among these research fields, indicating that interdisciplinary research can facilitate advancements in these areas in a more comprehensive and in-depth manner. Furthermore, the four clusters of “virulence,” “risk factors,” “elisa,” and “pcr” exhibit temporal continuity, implying that these areas are not only current hotspots but may also sustain their research vitality in the future, offering optimal solutions for the continued development of brucellosis research. The top three keywords in cluster #0 (virulence) highlight the primary focus of this cluster on immunity-related fields and mechanisms. The nodes exhibit more recent coloration, signifying significant research hotspot in recent years. Major risk factors for human brucellosis infection include occupational exposure, contact with aborted animals, consumption of meat, and ingestion of unpasteurized milk and raw cheese (3). Consequently, avoiding risks at the source is a preferable strategy for preventing brucellosis, although its implementation in epidemic areas is challenging (25). Therefore, preventive measures should persist while considering varying trends and the evolving epidemiology (26).

The analysis of keyword co-occurrence reveals that the burst value of “elisa” is the highest, at 41.47, indicating that this research area is highly representative and has garnered significant attention from researchers (27–29). Brucellosis has been the subject of study for over a century since its discovery, with research hotspots gradually shifting over time. Between 1990 and 2012, “elisa,”“monoclonal antibody” and “outer membrane protein” consistently ranked as the primary research hotspots in this field. However, since 2016, there have been notable shifts, with recent years focusing on areas such as “one health,” “risk factors” and “seroprevalence” that signify emerging frontiers and potential future directions in brucellosis research (30–32). An analysis of literature related to brucellosis published since 2024 indicates that 82.30% of the papers are research articles, with 56.14% utilizing natural hosts of *brucella* for studies that primarily analyze host seroprevalence, testing methods, infection mechanisms, integrated prevention and control, and vaccine research. Furthermore, 34.36% concentrate on naturally infected individuals, focusing on clinical features, diagnostic methods, treatment plans, differential diagnoses, prognosis, epidemiology, influencing factors, human seroprevalence, case reports, and laboratory testing methods. Additionally, 9.50% address meteorological environmental factors, alongside knowledge, attitudes, behaviors regarding brucellosis, and animal management. Review articles comprise 13.56% of the literature, primarily summarizing clinical treatment, pathogenesis, vaccination, and drug resistance, while 4.14% focus exclusively on bioinformatics statistical analysis. Consequently, current research is transitioning from an emphasis on traditional epidemiology, pathogenesis, testing methodologies, and vaccination toward a more integrated “one health” approach (33–36). This trend reflects a broader perspective among researchers, increasingly acknowledging the complex interactions among humans, animals, and ecosystems. Such an approach elucidates the direction for optimizing the prevention and control of brucellosis (37).

The integrated “one health” perspective applied to brucellosis research necessitates focusing on several key areas. First, regarding content, there is a growing incorporation of new terminologies into research hotspots, indicating that the scope and direction of brucellosis research are becoming increasingly diverse and gradually establishing a comprehensive, systematic research paradigm. Second, at the methodological level, continuous innovations in artificial intelligence technologies are enabling the deep integration and broad application of various technological advances in the management, detection, and prevention of brucellosis. This progress demonstrates significant developmental potential, facilitating the integration and precise extraction of multidimensional information. Third, in terms of value, the “one health” concept is gaining increasing attention and application. Effective management, detection, and prevention of brucellosis require interdisciplinary approaches, including environmental science, ecology, and zoology. Thus, multidisciplinary collaboration signifies that addressing brucellosis cannot rely solely on mechanisms, vaccines, and epidemiological studies; instead, it necessitates a comprehensive “one health” framework.

### 4.1 Limitations

This study has several limitations. First, the literature included in this study is restricted to the WoS Core Collection database, which may lead to incomplete literature inclusion, thus affecting the accuracy of the analysis results. Second, the citation frequency of literature requires time to accumulate, which may lead to lower citation volumes for recently published papers, thus limiting the assessment of their research contributions. Lastly, the nature of bibliometric analysis relies predominantly on co-citation, which may not fully reflect the quality of research; therefore, it is essential to incorporate clinical and methodological knowledge for a comprehensive interpretation of the research results (38, 39).

## 5 Conclusion

This study employed bibliometric methods to systematically review brucellosis research since 1901. The study found a rapid increase in the number of publications in this field since 2002, with research outputs concentrated in developed countries such as the United States, and Professor Pappas recognized as a core contributor in the field. Current research focuses on areas including pathogenic mechanisms, epidemiology, and detection technologies, while the “one health” concept is guiding future research directions. This quantitative analysis provides important references for optimizing disciplinary layout, advancing academic collaboration, and formulating public health policies in this field.

## Data Availability

The original contributions presented in the study are included in the article/supplementary material, further inquiries can be directed to the corresponding author.
